# Speeded Reaching Movements around Invisible Obstacles

**DOI:** 10.1371/journal.pcbi.1002676

**Published:** 2012-09-20

**Authors:** Todd E. Hudson, Uta Wolfe, Laurence T. Maloney

**Affiliations:** 1 Department of Psychology, New York University, New York, New York, United States of America; 2 Center for Neural Science, New York University, New York, New York, United States of America; 3 Department of Psychology, University of St. Thomas, Saint Paul, Minnesota, United States of America; 4 Program for Neuroscience, University of St. Thomas, Saint Paul, Minnesota, United States of America; University of Oxford, United Kingdom

## Abstract

We analyze the problem of obstacle avoidance from a Bayesian decision-theoretic perspective using an experimental task in which reaches around a virtual obstacle were made toward targets on an upright monitor. Subjects received monetary rewards for touching the target and incurred losses for accidentally touching the intervening obstacle. The locations of target-obstacle pairs within the workspace were varied from trial to trial. We compared human performance to that of a Bayesian ideal movement planner (who chooses motor strategies maximizing expected gain) using the Dominance Test employed in Hudson et al. (2007). The ideal movement planner suffers from the same sources of noise as the human, but selects movement plans that maximize expected gain in the presence of that noise. We find good agreement between the predictions of the model and actual performance in most but not all experimental conditions.

## Author Summary

In everyday, cluttered environments, moving to reach or grasp an object can result in unintended collisions with other objects along the path of movement. Depending on what we run into (a priceless Ming vase, a crotchety colleague) we can suffer serious monetary or social consequences. It makes sense to choose movement trajectories that trade off the value of reaching a goal against the consequences of unintended collisions along the way. In the research described here, subjects made speeded movements to touch targets while avoiding obstacles placed along the natural reach trajectory. There were explicit monetary rewards for hitting the target and explicit monetary costs for accidentally hitting the intervening obstacle. We varied the cost and location of the obstacle across conditions. The task was to earn as large a monetary bonus as possible, which required that reaches curve around obstacles only to the extent justified by the location and cost of the obstacle. We compared human performance in this task to that of a Bayesian movement planner who maximized expected gain on each trial. In most conditions, but not all, movement strategies were close to optimal.

## Introduction

Imagine that you are sitting at your desk with a nice, hot cup of coffee in front of you and your laptop keyboard roughly behind it. In reaching out to hit the return key, you plan a trajectory that takes into account the possibility that you might jostle the cup and spill your coffee – that is, you plan a movement trajectory that you would not pick if there were no coffee cup in the way. Whatever trajectory you pick, however, will typically deviate from the one that you planned due to noise/uncertainty in the neuro-motor system. This noise has two important consequences: a risk of inadvertently spilling your coffee, and a risk of missing the key altogether. Your choice of plan involves a tradeoff between the costs and rewards associated with the possible outcomes of your planned movement.

The motor system, in planning any speeded movement, is selecting a stochastic “bundle” of possible trajectories [1], [2] and the particular bundle chosen determines the probabilities of favorable and unfavorable outcomes. There is no basis for selecting one planned trajectory as “best” without knowing the consequences of these different outcomes. If you are reaching to prevent your laptop from deleting your morning's work, you may be quite willing to put your coffee in peril and clean up later. In this article, we consider the problem of obstacle avoidance within the framework of Bayesian decision theory.

In this first investigation of obstacle avoidance within the framework of Bayesian decision theory, we translate the above example to one where there is an explicit reward for touching targets and an explicit cost for inadvertently intersecting intervening obstacles. We examine human obstacle-avoidance reach trajectories relative to the benchmark performance of an optimal Bayesian reach planner that chooses motor strategies to maximize expected gain as described next.

### The Experimental Task

**Figure 1:**
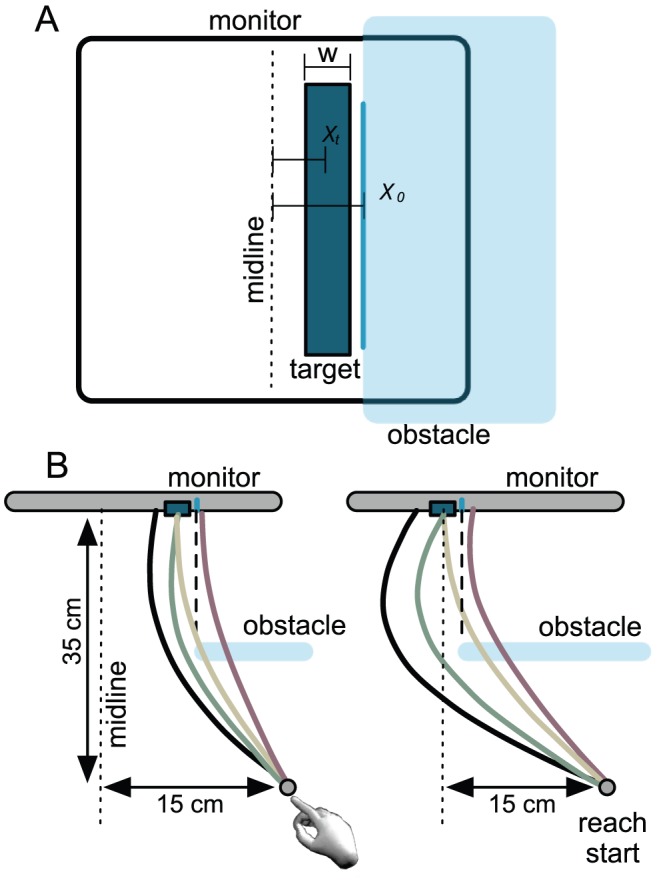
Planning and reaching with obstacles present. The subject attempts to touch a target on a computer screen while avoiding an invisible obstacle placed partway along the trajectory of movement that the subject would take if the obstacle were not present (shown as a transparent blue plane in these figures). **A.** From the subject's viewpoint. **B.** Two examples, seen from above, with possible trajectories marked. Both target and obstacle are elongated vertically so that only the horizontal *x*-coordinates of possible movement trajectories (shown as colored traces connecting start position and monitor screen) affect the resulting rewards and costs. The obstacle edge was separated from the target by 6.6 mm in all conditions (i.e., 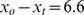 mm).

The experimental task illustrated in Figure 1 contains many of the elements of our coffee-cup example, and is reminiscent of the kind of obstacle avoidance behavior that has been studied extensively both in terms of its neurophysiological substrates [3], [4], [5] and in identifying sensory/motor factors that influence the movement trajectory [6], [7], [8], [9], [10], [11], [12], [13]. We will describe it in detail in the next section.

To study obstacle-avoidance reaches within the framework of Bayesian decision theory, we translated the above example to one where there is an explicit reward (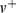) associated with touching a target and an explicit cost (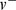) associated with inadvertently intersecting an obstacle that is placed between the starting point of the hand and the target. Contact with a physical obstacle placed along the reach path might change the physical character of the reach and such an obstacle would constitute an intrinsic cost whose value we could not easily measure or manipulate. To avoid these issues, we used virtual obstacles that could not impede the reach.

Although the virtual obstacle is invisible, a visual indication of its leftmost edge (at 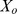) is presented on the monitor prior to each reach. Figure 1A shows a front view of the experimental apparatus with the virtual obstacle shown in transparent blue. The blue line on the monitor marks its edge (at 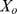). The subject incurs the cost 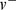 if the fingertip passes through the virtual obstacle while reaching toward the target (centered on 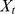, with width 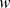). One part of training will allow subjects to become familiar with the location of the obstacle in depth and how its edge relates to the visual marker (the blue line). Across experimental conditions we varied the location of the obstacle 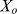 and target 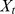 and the cost 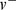 incurred by passing through the obstacle as described in the next section. In all conditions there was a constant relative distance between the obstacle edge 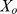 and the center of the target 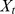. Figure 1B show the same setup but from an overhead viewpoint. The left and right panels differ in the location of the obstacle-target pair.

#### Notation

Reward on each trial is determined by (a) the point where the fingertip passes through the fronto-parallel plane containing the obstacle and (b) where it contacts the fronto-parallel plane containing the target. By making the target a vertical strip and the obstacle region a half-plane with a vertical edge, we reduce the analysis of data to observations in the horizontal dimension. In the horizontal dimension, a pair of points in the obstacle and target planes is given by the coordinate 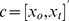, where horizontal coordinates increase from left to right. The same coordinate given relative to the obstacle edge and target center is 
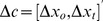
 with 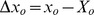 and 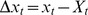. We refer to 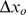 as the fingertip *excursion* around the obstacle; i.e., the extent to which the fingertip avoids the obstacle edge. When we average excursions within an experimental condition we refer to the average 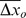 as the average excursion and 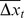 as the average endpoint. The subject incurs a cost if and only if he passes to the right of the obstacle edge (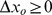) and earns the reward if and only if he hits within the target (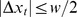).

There are four possible outcomes (illustrated in the Figure 1B,C), denoted 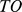 (hit target and obstacle), 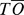 (hit target, miss obstacle), 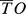 (miss target, hit obstacle), and 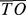 (miss target, miss obstacle). Figure 1B and Figure 1C differ in the placement of the target (and therefore also the obstacle).

#### Expected gain

**Figure 2:**
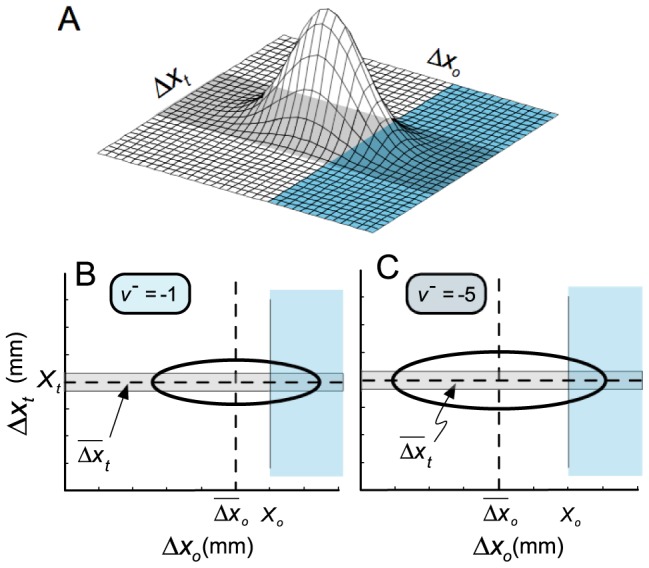
Value diagrams. **A.** Probability distribution overlaid on a value diagram. For any speeded trajectory past the obstacle to the target, the actual trajectory will differ from the trajectory plan due to motor noise inherent in speeded movement. There are two critical points along the actual trajectory that determine the rewards and costs derived from each movement: the depth planes containing the obstacle and target. We denote the horizontal excursion of the fingertip from the edge of the obstacle (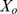) and the center of the target (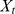) within their respective depth planes as 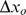 and 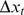. Value diagrams plot the 2D space of 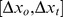 coordinates, with obstacle and target regions colored in blue and grey (respectively). Reward and cost regions overlap when reaches intersect both obstacle and target. We superimpose the probability distribution induced on 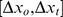 when the subject attempts to execute a particular speeded trajectory. Any planned trajectory induces such a probability distribution, and the subject in planning is effectively choosing among possible distributions. The probability volume over the blue region defines the probability of hitting the obstacle, and the probability volume over the grey region defines the probability of hitting the target. **B**. Value diagram with the probability distribution representing possible intersection-points at the two planes schematized as an equal-probability ellipse. When the obstacle cost is small, a trajectory plan passing relatively near to the obstacle may be chosen. The choice of trajectory potentially affects the covariance of 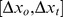 and the probabilities of hitting targets and reward. **C.** For larger obstacle costs subjects might choose a larger trajectory excursion to avoid the obstacle. Again, the choice of planned trajectory potentially affects the covariance of 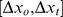, which we have here drawn as noisier overall and particularly so in the 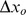 dimension. In the experiment we will model the effect of changing excursion on covariance and use this model to predict the planned trajectory (and distribution) that maximizes expected gain for any choice of cost function.

Both 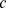 and 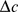 are 2D coordinates. In Figure 2A we plot a hypothetical bivariate Gaussian distribution on 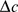 and label the region that corresponds to an obstacle in blue and the region that corresponds to the target in grey. These regions overlap since it is possible to touch both the obstacle and target on a single trial. We refer to this plot as the *value diagram*.

On each trial the subject selects and executes a movement plan or motor strategy 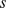, and this strategy determines the distribution 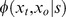 on 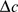. The expected gain associated with a given motor strategy is
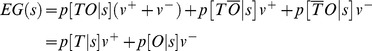
(1)We can induce changes in the distribution shown in Figure 2A by manipulating the locations and costs of the obstacle (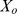) and target (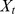). The two unknown terms of (1) are computed as follows:
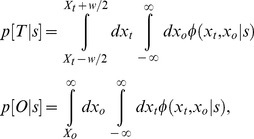
(2)where the 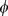 distribution is defined by
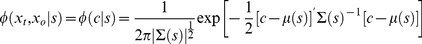
(3)with 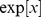 denoting 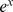.

Equation (3) is the probability density function of a bivariate Gaussian (see Supplemental Figure S1) determined by choice of motor strategy 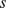. The 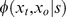 distribution describes deviations from the planned trajectory as it intersects the two critical planes defined by our experiment. It is parameterized by the planned (theoretical) intersection coordinates 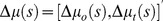 and covariance matrix 
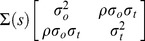
, whose elements are functions of 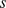. To simplify notation, we will sometimes omit 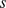, writing 
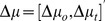
, 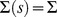, etc.

When the subject chooses a planned trajectory he effectively chooses the *planned coordinates* 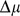 at which the planned trajectory intersects the obstacle and target planes. As suggested in Figures 1 and 2 we expect that the subject will plan excursions 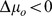 to the left of the obstacle and endpoints 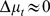 centered on the target, and as a consequence 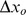 will typically but not always be less than 0 (to the left of the obstacle).

#### A hypothetical example

In Figure 2B,C we illustrate the hypothetical effect of increasing the magnitude of 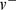 from −1 to −5. The ellipses represent the covariance 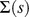 of the bivariate Gaussian 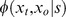. In response to the change in cost, the subject moves the average excursion point away from the edge of the obstacle and the covariance ellipse 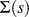 grows. Intuitively, the hypothetical subject has chosen a larger excursion (i.e., a more curved trajectory around the obstacle) in response to the increase in cost and the intersection of the trajectory with the obstacle plane 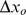 becomes more variable. While the example is hypothetical, we will see similar patterns in human performance.

#### The Dominance Test

Our goal is to examine human obstacle-avoidance reach trajectories relative to the benchmark performance of a Bayesian movement planner that chooses the movement strategy 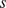 maximizing expected gain (Equation 1) for any choice of target and obstacle location and any choice of cost and reward. If we knew the full range of possible movement strategies 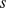 we could calculate the strategy (or strategies) 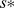 that maximized the subject's expected gain in any given experimental condition. We could then compare the subject's average trajectory in each condition to the theoretical trajectories predicted by the model. Any discrepancy between measured performance and predicted would be sufficient to reject the Bayesian model. Of greatest interest, though, would be evidence of patterned deviations from optimality. If, for example, the subject consistently picked excursions 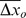 in all conditions farther from the obstacle plane than the predicted optimal 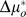 for that condition, then we could interpret the subject's failure as a kind of loss aversion [14]: the subject is giving too much weight to avoiding the obstacle.

The key problem in comparing human performance to a Bayesian model maximizing expected gain is that we have no theoretical model of the possible trajectory bundles available to the subject even in the simplest reaching movement. One solution is to build an empirical model based on observed movement strategies under a range of experimental conditions; that is, to measure the possible types of trajectory bundles that might be produced. One can then determine the optimal movement strategy for each condition based on that empirical model.

Hudson et al. [15] formalize this approach as a *Dominance Test*. It consists of two stages: First, characterize the possible movement strategies available to the subject under the conditions of the experiment by analyzing the subject's performance. Second, test whether, in each experimental condition, the subject selected the movement strategy available to him that maximized expected gain. Suppose, for example, that the subject chose movement strategy 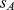 in condition A of an experiment, strategy 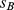 in condition B, and so on through condition D. One can then calculate the expected gain that the subject could achieve by applying strategy 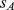 through strategy 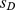 in condition A.

Now suppose that, for example, the gain that would result from applying 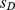 in condition A is the maximum of the gains expected from applying 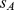 through 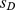 in condition A. In particular, it is greater than the expected gain resulting from applying 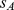 in condition A. We say that 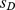 “dominates” 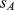, 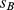 and 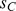 in condition A, and, in this hypothetical example, we can be certain that the subject failed to pick the strategy maximizing expected gain in condition A because a strategy known to actually be exhibited by the subject (in condition D) would have performed better in this condition (condition A).

Hudson et al. [15] apply the Dominance Test in each of two experiments and, in the second, reject the hypothesis that subjects maximized expected gain. They found that subjects consistently selected movement strategies that were too “slow”. We develop a similar approach to testing optimality for the conditions of the experiment reported here.

#### Modeling assumptions

The evident complexity introduced by the obstacle is that the covariance term 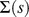 as well as the desired trajectory 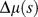 change as we change motor strategy and we must develop models of how 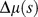 and 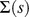 change, based on the reach trajectories observed in our experiment. We make two simplifying assumptions, both of which we will verify in the data. First, 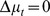; the subject aims for the center of the target. Second, for any choice of obstacle location 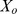, the choice of planned excursion 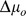
in the obstacle plane determines both the standard deviation in the obstacle plane (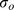) and in the target plane (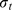). The result is an empirical model of the trajectory bundles (i.e., the distributions 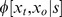) available to the subject.

The novelty of our approach is threefold: (1) We are examining the tradeoff between uncertainty at two points along a reach trajectory, manipulating this tradeoff by altering the costs associated with intersecting the obstacle. (2) We are considering “soft obstacles” where, given an appropriate cost structure, the optimal choice of movement plan may involve a high risk of hitting the obstacle. (3) We apply a method that allows us to compare human obstacle avoidance to the predictions of a Bayesian model even when we have no theoretical model of the possible trajectory bundles available to the subject (the Dominance Test).

## Materials and Methods

### Ethics Statement

Seven naive subjects participated in the experiment. Subjects were paid for their time ($10/hr.) and also received a bonus based on points earned during the experiment that amounted to $.01 per point (an additional $5–$10 over the hourly rate). All participants provided informed consent and research protocols were approved by the local Institutional Review Board.

### Apparatus

Subjects were seated in a dimly lit room 42.5 cm away from a fronto-parallel transparent polycarbonate screen mounted flush to the front of a 21″ computer monitor (Sony Multiscan G500, 1920×1440 pixels, 60 Hz). Reach trajectories were recorded using a Northern Digital Optotrak 3D motion capture system with two three-camera heads located above-left and above-right of the subject. Subjects wore a ring over the distal joint of the right index finger. A small (0.75×7 cm) wing, bent 20 deg at the center, was attached to the ring. Three infrared emitting diodes (IREDs) were attached to each half of the wing, the 3D locations of which were tracked by the Optotrak system. Further details of the apparatus are given in a recent report [16]. The experiment was run using the Psychophysics Toolbox software [17], [18] and the Northern Digital API (for controlling the Optotrak) on a Pentium III Dell Precision workstation.

### Stimuli

Subjects attempted to touch targets on a computer screen, represented visually as a vertical [6.5 mm×15 cm] strip, whose locations were chosen randomly and uniformly from a set of three locations [0, 38, 75 mm] relative to the monitor center. Rewards and penalties were specified in terms of points. Hits on the target earned subjects two points, and passing through the obstacle incurred a cost of one, two or five points. Missing the target earned no points, and too-slow reaches incurred a cost of ten points.

### Task

*All reaches*. All trials proceeded as follows: subjects brought their right index finger to a fixed starting position at the front edge of the table (15 cm to the right of screen center), triggering the start of the trial. Next, the target (and obstacle) was displayed (Figure 1A), followed 50 ms later by a brief tone indicating that subjects could begin their reach when ready. Movement onset was defined as the moment the fingertip crossed a frontal plane 3 mm in front of the table edge, itself located 35 cm from the screen; the fingertip was required to reach the screen within 600 ms of movement onset. Both the fingertip endpoint, the location where the fingertip passed through the plane of the obstacle (during obstacle practice and experimental reaches) and a running total of points (during experimental reaches) were displayed on-screen at reach completion.

### Procedure

#### Target practice

Subjects were first given practice making reaches to targets on the screen. Targets were selected randomly from the set of three target locations, with 50 of each target presented. During target practice no points were awarded, and no obstacles were present.

#### Obstacle practice

Following practice reaching to the three target locations, subjects were given an opportunity to learn the location of the *obstacle plane* in 3D space along the reach path. The obstacle always occupied part of the plane at the halfway point between the reach start and the monitor (parallel to the monitor). A vertical line representing the obstacle edge and a small circle near that vertical line were drawn on the screen. The vertical line represented the leftmost edge of the obstacle, and the circle was the target to be touched during obstacle practice. Subjects did not attempt to hit the target by touching the screen. They were instructed to make a “poking” motion in the air in front of targets presented onscreen. When the fingertip passed through the *obstacle plane*, a ‘click’ was played; a dot at the corresponding screen location was also drawn – in blue if the fingertip passed through the *obstacle* (to the right of the vertical line), and in grey otherwise. When the fingertip crossed the obstacle plane within the target, the onscreen representation of the target “exploded”, indicating that the target had been successfully touched. The set of vertical lines was chosen randomly from a uniform distribution extending over the set of obstacle edges used in the main experiment (see below), and target positions were chosen randomly from a second uniform distribution to fall within 1.5 cm (horizontally) of the vertical line. After 50 targets in the obstacle plane had been successfully touched, the main experiment began.

#### Main experiment

There were two differences between reaches to onscreen targets during target practice and reaches in the main experiment. First the virtual obstacle, whose leftmost edge was always located 6.6 mm to the right of the target, was present. And second, a running total score, along with feedback concerning whether target, obstacle or both had been touched, were given at the end of each movement. The three possible target locations 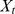 were 0 mm, 38 mm and 75 mm to the right of the center of the screen. The three target locations 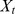 combined with three obstacle values 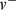 at each target for a total of nine experimental conditions. Conditions were blocked, such that each condition occurred four times during the experiment, and 30 reaches were performed in each block, for a total of 1080 reaches. An instruction screen appeared at the start of each block indicating the values 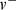 and 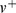.

### Data Collection

Before each experimental session, subjects (fitted with IREDs) touched their right index finger (pointing finger) to a metal calibration nub located to the right of the screen while the Optotrak recorded the locations of the six IREDs on the finger 150 times. Linear transformations converting a least-squares fit of the three vectors derived from the 3 IREDs on each wing (left and right; each defining a coordinate frame) into the fingertip location at the metal nub were computed.

During each reach we recorded the 3D positions of all IREDs at 200 Hz and converted them into fingertip location using this transformation. The 3 IREDs on the left and right wings were used to obtain fingertip location independently, and the two estimates were averaged when all IRED locations were available for analysis. This redundancy allowed data to be obtained even if IREDs on one wing or the other were occluded during some portion of a reach.

### Modeling Optimal Reach Plans

Because we cannot predict the biomechanical costs associated with reach speed and overall length of reach trajectory that might accompany the longer and faster reaches necessary to reach targets within the timeout interval for, e.g., midline vs. right-of-midline target locations, we restrict the cost function that must be minimized by an optimal reach planner to the target and obstacle costs defined by 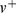 and 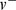. Thus, the only factors entering into the optimal reach plan are fingertip positional uncertainty (i.e., the standard deviation of fingertip position in the relevant plane), average fingertip coordinates at the two critical planes, 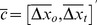, and target and obstacle costs. To compute optimal reach plans, we model the empirical relationship between mean excursion, 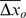, and the remaining kinematic variables, the two sample standard deviations, 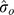 and 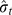 at the obstacle and target planes, respectively. The relationships were close to linear and we thus fit three lines relating empirical fingertip standard deviation 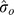 to mean excursion 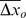 separately for each of the three obstacle positions 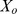 because we allowed for the possibility that fingertip standard deviations will change differently for excursions around nearby and further-away obstacles. Similarly we fit three lines relating empirical fingertip standard deviation 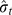 to mean excursion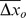. These six lines allowed us to predict 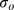 and 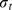 as a function of any planned excursion 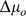. While it is plausible that we could develop a single equation to predict each of the standard deviations, 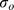 and 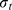 by incorporating the obstacle location 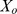 itself we could only do so at the cost of additional assumptions; the equations we use are sufficient for our purposes.

After having obtained a function relating excursion size and fingertip uncertainty (at both the target and obstacle planes, for all three obstacle positions), it is possible to predict fingertip standard deviations for theoretical excursions (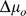) not observed experimentally, around any of our obstacles. This in turn allows one to compute the expected gain associated with any theoretical excursion. Maximizing the expected gain function yields the prediction of the optimal reach planner (i.e., the theoretical excursion maximizing expected gain, 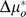) in each of the 9 conditions of the experiment.

### Statistical Analysis

In the previous section we outline our method of predicting the obstacle avoidance behavior of an optimal Bayesian reach planner based on modeled changes in uncertainty, both at the obstacle plane and the target plane, of making reaches that deviate from their natural unobstructed trajectory. Because we parameterize the expected gain function in terms of obstacle-plane excursion, we can test the hypothesis that data conform to the predictions of the optimal Bayesian reach planner by comparing predicted 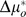 and observed (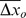) obstacle-plane excursions. Data conforming to the Bayesian (optimal planning) model will fall along the identity line of a plot showing observed vs. predicted excursions.

Notice that we manipulated value to get the range of data needed to predict the standard deviations 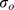 and 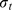 given the planned excursion 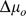, and we then use these equations to predict the optimal excursion 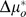 for each condition. The reader may be concerned that there is an apparent circularity in our use of the Dominance Test. The circularity is only apparent, not actual; This is because, no matter how well the empirical fits (relating planned excursion to standard deviations 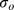 and 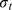) fit the data, there is no guarantee that the average excursion (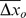) observed in a particular condition, of all possible excursions, will produce the largest possible gain; i.e., that it happened to fall at the theoretical MEG excursion (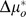) for that condition. Suppose, for example, that the subject consistently chose excursions that are 80% of the way between the edge of the obstacle and the theoretical MEG excursion (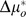). While the observer has failed to maximize expected gain in *every* condition, the fits relating planned excursions to standard deviations 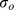 and 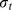 will be little affected. We refer the reader to the second experiment of Hudson et al. [15], which used a similar Dominance Test and demonstrated such a patterned failure.

We compare performance to that predicted by the optimal planning model using standard Bayesian model comparison techniques (see Supplemental Text S1). This analysis yields a *measure of evidence* [19] (given in decibels) for the optimal planning model (or conversely, against non-optimal planning models), based on the odds ratio comparing the probability of the optimal planning model given the observed data and the probability of any of the non-optimal models on the same data. For example, evidence of between 3 and 4.75 dB (or odds of between 2: and 3∶1 favoring one model over the alternative[s]) is usually considered a lower bound for statistically significant evidence [see e.g.], [ 15], [16], [19], [20,21].

## Results

### Value Diagrams

**Figure 3:**
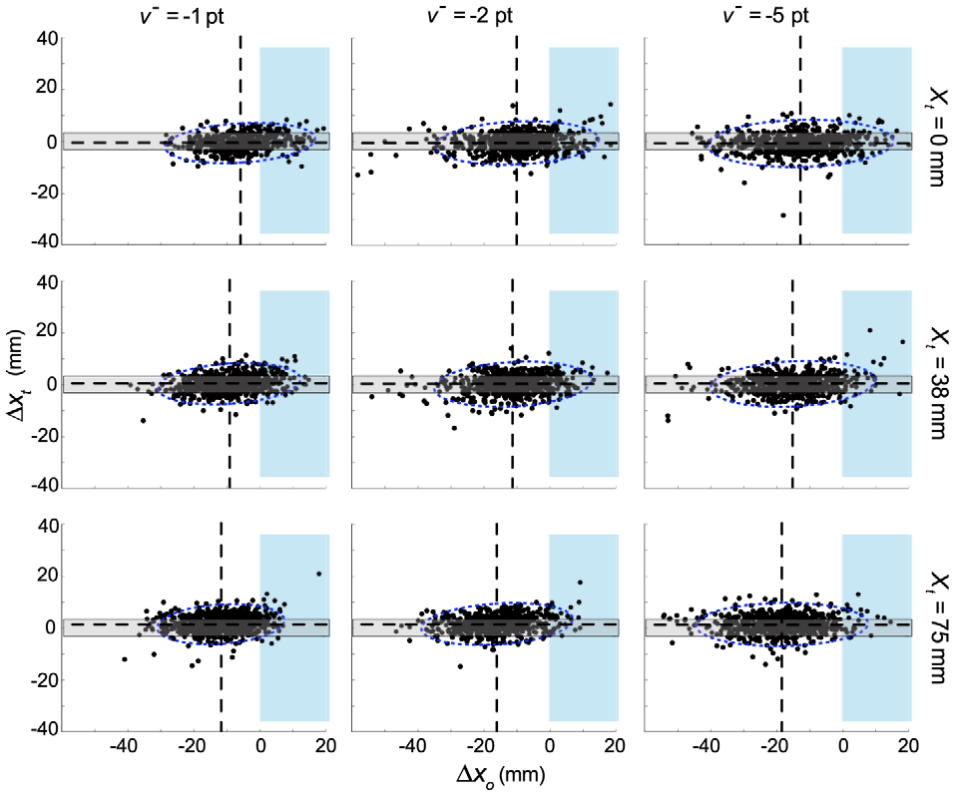
Value diagrams for three obstacle costs and three obstacle positions. Each value diagram plots the horizontal excursion from the edge of the obstacle 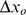 on the horizontal axis and the horizontal error from the center of the target 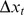 on the vertical axis for all subjects. Individual subject data corresponding to a given condition are plotted centered on the pooled (across subjects) mean, 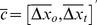. Each column of value diagrams corresponds to a different obstacle cost (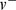), and each row of diagrams corresponds to a different target/obstacle pair (given in terms of 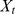, relative to the center of the monitor).

**Figure 4:**
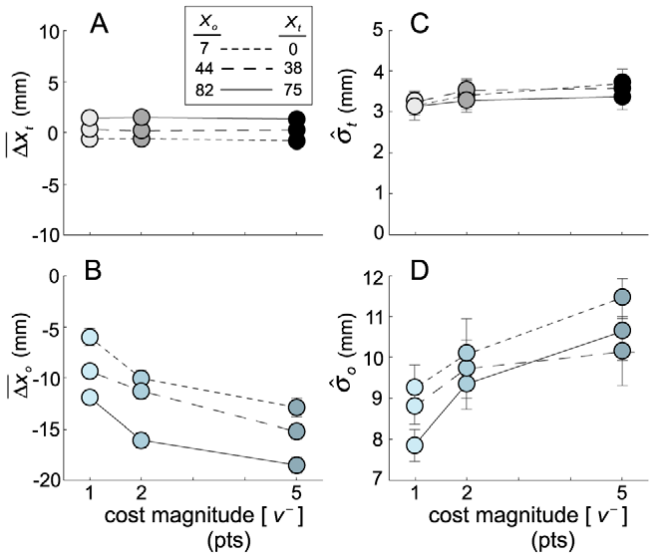
Summary data. **A.** Horizontal target-relative endpoint errors 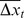 as a function of obstacle cost. Although cost magnitude has little effect, average errors vary slightly with target position. **B.** Average fingertip excursions 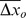 as a function of obstacle cost. Trajectories move further from the obstacle (larger 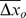 magnitudes) with increasing obstacle costs, and with increasing distance of the obstacle from the screen center. **C.** Standard deviation of pooled target plane errors 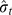 as a function of obstacle cost for each target position. Standard deviations increase with cost magnitude. **D**. Standard deviation of pooled fingertip excursions 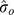 as a function of obstacle cost for each obstacle location. Standard deviations increase with cost magnitude. All errors bars are ±1 standard error of the mean.

Several features of the data can be observed directly in the value diagrams (Figure 3). First, higher costs lead subjects to avoid the obstacle region by a greater margin: there is an increasing deviation between obstacle-plane crossing points and the obstacle edge as 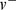 magnitudes increase, across all targets. However, this change in crossing-point is not accompanied by within-target changes in average target-relative endpoints: no matter how great an excursion the finger took around the obstacle, the location of the distribution of target endpoints was unchanged. This relation of endpoint error with target position alone (i.e., independent of excursion) allowed us to model 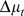 as the average endpoint error in each condition (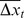), regardless of excursion size. In addition, covariance ellipses consistently increase in size as 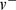 magnitudes increase (within each target location). These four functions, relating changes in positions and standard deviations to 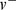 magnitude, are plotted in Figure 4.

One can also see a slight positive correlation (“counterclockwise tilt”) in value diagram covariance ellipses (Figure 3). That is, a rightward deviation from the mean in the obstacle plane tends to be paired with a rightward deviation in the target plane. This correlation implies that there is a component of the trial-to-trial variation in trajectories that affects the entire reach, and is therefore detectable at both obstacle and target planes. This tendency is quite small, however, and is ignored in our modeling.

### Modeling Covariance

We have developed a simple empirical model of the relationship between horizontal excursion within the obstacle plane and horizontal variance. While the model allows us to predict optimal behavior, we make no claims regarding the factors affecting horizontal variance.

Our study was not designed to determine the origins of positional uncertainty, a separate and intriguing question. There are very likely many factors that contribute separately to sensory and motor uncertainty and we implicitly assume that those factors (in our task, direction of gaze, body posture, etc.) are selected by the visuo-motor system so as to provide the best possible tradeoffs between hitting the target and avoiding the obstacles.

**Figure 5:**
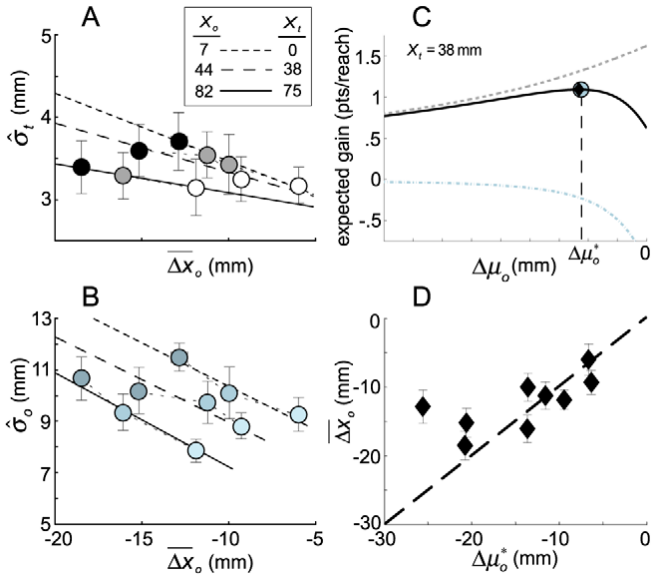
Planned trajectories maximizing expected gain. **A.** Plot of 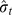 versus 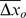 for each obstacle position 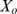 and cost 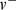 (grey level). Error bars are ±1 standard error. Lines show weighted least-squares fits of the variation of 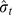 with mean excursion in the obstacle plane 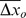 for each obstacle location across subjects. **B.** Plot of 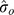 versus 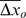 for each obstacle position 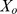 and each cost 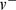. Error bars are ±1 standard error. Lines show weighted least-squares fits of variation in 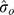 with mean obstacle plane excursion (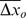) at each obstacle location across subjects. In panels A and B, increasing cost magnitudes correspond to darker symbol shading. The fits in these panels represent our estimates of the linear functions relating excursion size 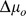 to standard deviations 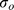 and 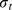 in the two critical planes. **C.** An example of how expected gain varies as a function of theoretical excursion 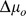 for one experimental condition. Panels A and B form the basis for predictions of covariance changes as a function of any planned excursion (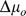) in each experimental condition, which in turn allows for prediction of the effect of 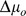 on expected gain. As obstacle plane excursions 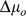 decrease (the trajectory moves closer to the obstacle), the probability of hitting the obstacle increases and the expected cost (plotted as a blue dot-dashed curve) magnitude increases. At the same time, the probability of hitting the target increases and the expected reward incurred by hitting the target increases (grey dashed curve). The expected gain, the sum of the expected cost and expected reward, is the solid back curve that attains its maximum (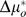) at the location of the blue dot. For comparison, the mean excursion across subjects for this condition (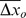) is plotted as a black diamond. **D.** A plot of the observed average excursion (±1 standard deviation) in the obstacle plane 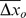 (averaged across subjects) versus the optimal shift that would maximize expected gain 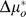 at each of the nine experimental conditions.

To compute optimal reach plans based on the data available in the value diagrams, we re-organize the plots in Figure 4 to predict target- and obstacle-plane fingertip positional uncertainty as functions of the observed obstacle-relative fingertip excursion (Figures 5A and 5B, respectively). Fitting straight-line functions to these data by linear regression (i.e., a line was fit to the data from each obstacle condition separately; *R* ^2^ ranged from 0.8 to 0.99), we can predict target- and obstacle-plane uncertainties at unobserved fingertip excursions. By varying the theoretical planned excursion (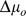), we compute the expected gain (Equations 1–3) at the obstacle plane (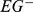), the target plane (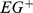) and overall, predicted as a function of any possible (i.e., non-positive) planned obstacle-plane excursion for each obstacle location and 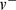 magnitude. An illustration of the computation is given in Figure 5C, corresponding to the middle target location and the middle obstacle cost. The maximum of the expected gain curve as a function of theoretical excursion, 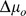, corresponds to the excursion in the obstacle plane that maximizes expected gain, denoted 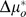.

The mean observed excursion 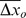 across subjects is plotted versus the excursion maximizing expected gain 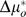 in Figure 5D. The confidence intervals are 95% confidence intervals across subjects. An optimal reach planner would produce data along the identity line of this plot. Overall, the Bayesian evidence measure we computed is 12.99 dB (about 20∶1 odds) favoring the hypothesis that data do, in fact, fall along the identity line. However, there are deviations when the predicted MEG excursion (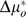) is large in magnitude (leftmost point in Figure 5D) where the mean observed shift is almost a factor of two smaller than the predicted shift. While human performance for smaller excursions is not far from optimal, there is a clear failure of optimality for the largest predicted excursion. Subjects passed too close to the obstacle in following their trajectory to the target.

### Stationarity

The optimal reach planning model described here assumes that the distribution 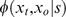 on 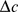 is stationary (does not change across time). We considered the possibility that subjects might employ a within-block “hill-climbing” strategy designed to discover the MEG excursion by initially making too-large excursions around the obstacle and reducing their size over the following few reaches until an appropriate point was found. We verified that this was not the case in the Supplement (Supplemental Figure S2). There, we show that the distribution of excursions 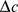 does not vary appreciably over the course of each block. To further investigate the possibility of similar cognitive strategies, we computed autocorrelations for each subject and block up to lag 15. No significant autocorrelations were found, suggesting that cognitive “contamination” was not present in our results.

## Discussion

We developed a model of obstacle avoidance within the framework of Bayesian decision theory and tested that model experimentally. We considered the possibility that reach trajectories around an obstacle can be explained quantitatively by a reach planner that minimizes the overall negative effect of an intervening obstacle. Such a reach planner would optimize the trade-off that increases excursion extent to reduce the expected cost of contacting the obstacle, but also decreases excursion extents so that the probability of contacting the eventual target is not drastically reduced.

This work represents a different approach to the problem than is traditionally taken: We are not attempting to determine how specific elements of the display determine changes in the details of the obstacle-avoidance reach or affect the possible covariance structures at the two points along the trajectory of interest. The Bayesian decision-theoretic approach [22], [23], [24], [25], [26] allows us to model and consider a wider range of tasks, of which simply hitting the target or avoiding the obstacle are at the extremes of a continuum. We frame the problem as a tradeoff among possible value-weighted outcomes with the motor system able to select among movement plans that assign probabilities to those outcomes [15].

We focused on a task where the key tradeoff is between the uncertainties at two locations (depth planes) along a reach trajectory, and we examined the covariance structure induced by a virtual obstacle placed between the subject and the goal. We employed a method for testing whether subjects maximize expected gain (the Dominance Test) based on an empirical characterization of relevant movement strategies available to the subject followed by a test, in each experimental condition, of whether the subject has selected the movement strategy that maximizes expected gain.

Studies aimed at identifying the visual [e.g.], [ 7,11], proprioceptive and biomechanical [e.g.], [ 8,27] elements that affect the specific form of a reach around an obstacle provide valuable contributions to solving the engineering problem of *how* these variables interact to modify reach trajectories planned around an obstacle. Our goal was different. We asked *why*, out of all possibilities, reach trajectories during obstacle avoidance have the form that they do.

### Value Manipulation

Reaches have goals. Although particularly obvious when reaching around an obstacle, this aspect of reach planning in the presence of an intervening obstacle has previously been ignored. This has created something of a dilemma for subjects, who must choose how much ‘weight’ to assign to accidentally contacting an obstacle vs. successfully touching the target (reminiscent of studies where one is instructed to perform a task ‘as quickly as possible without sacrificing accuracy’). Subjects must resolve the conflict created by these contradictory goals by choosing a relative weighting, a weighting that cannot generally be inferred from the data alone. Here, we avoid these problems; obstacles are assigned a cost, giving a clear indication of the relative ‘importance’ of accidentally contacting an obstacle and of contacting the reach target.

Not only does our value manipulation allow us to avoid the uncertainty associated with arbitrary target and obstacle weightings that change by subject (and possibly by experimental condition), it is also a necessary element of an optimal model of obstacle-avoidance reach trajectories. The value component of (1) allows us to quantitatively predict the excursion magnitudes that form the basis of the comparison shown in Figure 5D. This in turn allows us to separate the optimal planning model (data on the unity line of Figure 5D) from other models of trajectory planning around the virtual obstacle that might make the same qualitative predictions, but are nevertheless quantitatively sub-optimal (though not observed, such data would lie along a non-unity-line in Figure 5D). Such a separation between qualitative and quantitative optimal performance is demonstrated in Tassinari et al. [28] and in Hudson et al. [15].

### Optimal Feedback Control

Our data have implications for a class of popular models of obstacle avoidance and reach planning in general based on optimal linear feedback control [29], [30], [31]. One important prediction of these models is that 2D and 3D variance may be partitioned among the axes to produce the best task performance; for example more precision may be required along the horizontal than along the vertical dimension, as in the current experiments. Such a system is capable of partitioning more variance to the dimension requiring less precision. Here, for the first time, we are looking at a task where variance at two points along the trajectory of a reaching movement affects the outcome of the movement. We find no evidence that subjects partition their covariance in response to rewards or costs. Had they done so, there should have been increased vertical variance, not increased horizontal variance. That is, any manipulation that in fact increased horizontal variance should have been ‘referred’ to the vertical dimension, where it would not have adversely affected performance.

### Trajectories Outside the Obstacle and Target Planes and Multiple Trajectory Constraints

We confined analysis to the intersection of trajectories with the obstacle and target planes. The subject's reward is determined by these two points: fingertip position at the intersection of the obstacle and target plane, nothing more. The subject should select a movement plan, 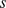, with the criteria that means and covariances in passing through these two critical planes maximize expected gain. Movement plans that satisfy these criteria clearly form a subset of all possible plans, but are they unique? Does the choice of the movement plan that maximizes expected gain in our experiment determine the entire trajectory bundle? Or, are there multiple planned trajectories (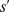,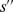, etc.) that match 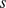 in mean and covariance at the two critical planes, but that deviate from 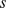 elsewhere? We cannot exclude this possibility nor can we exclude the possibility that a subject chooses now 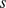, now 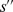, now 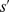, as he pleases. All would count as optimal choices of movement plan. The constraints we impose in the obstacle and target planes serve to select a set of equivalent optimal movement plans but further research is needed to determine the effect of the constraints we impose on the trajectory outside of the obstacle and target plane. In particular we avoided using data from outside the obstacle and target plane precisely because measured means and covariances at points along the trajectory outside of the obstacle and target planes may not reflect any single movement plan and it would be inappropriate to analyze them as if they were determined by the constraints of our task.

In our task the location of the fingertip at just two points along the trajectory determines the resulting reward or cost. We can readily generalize the task by adding additional obstacles along the path to create tasks for which the subject must consider his covariance at many points along the trajectory. This sort of generalization would allow investigation of the possible covariance structures along the reach trajectory available to the motor system. It also serves as a model task mimicking the constraints of many natural tasks where the goal is to maneuver around multiple obstacles to reach a goal, as in reaching into a computer chassis to extract one component.

### Biological Costs

We found that subjects' performance was close to that of a Bayesian decision-theoretic movement planner maximizing expected gain except for the most extreme conditions where the optimal choice of trajectory required a large excursion (“detour”) around the virtual obstacle. One possible explanation is that such movements entail a large biological cost and that the subject includes biological costs in the computation of expected gain. In effect he “prices” biological cost and is willing to reduce his monetary gain in order to reduce biological cost as well (see discussion in [32]). Although our current data cannot speak to this possibility, one might predict that separate measurements of biomechanical cost would allow these extreme conditions to be predicted as well.

The costs in our task are monetary but in theory would also apply to tasks where movement constraints are the results of injury or disease to the motor system [33], [34]. Patients might limit their motor repertoire in order to prevent undesirable outcomes such as pain or clumsiness, leading to long-term, conditioned motor deficits. This idea forms the basis of a now well-established rehabilitation approach, Constraint Induced Movement Therapy, in which the reward/cost structure of the environment is manipulated in ways that encourage the use of the previously avoided regions of motor space [35].

The conclusions we draw are based on movements confined to a narrow, clearly visible region of space immediately in front of the reviewer. Subjects presumably have considerable experience in coordinating eye and hand in this region of space before they begin the experiment. It would be interesting to investigate in future work with a full range of arm movements, including whether movement plans tend to avoid awkward or unusual movements.

### Summary

We examined the problem of obstacle avoidance from the standpoint of Bayesian decision theory. Our approach is different from other work in the area of obstacle avoidance. Previously, this problem has been approached from the standpoint of theories that suggest that the CNS minimizes kinematic or dynamic variables (e.g., total force production), with the constraint that the hand path not intersect an obstacle. Of course, this approach fails to take account of two major contributions to real-world movement plans: the uncertainty of visual estimates and motor outcomes (even for the same real-world obstacle and planned trajectory), and variable costs associated with intersecting different kinds of obstacles (accidentally toppling a cup of water is very different from toppling a cup of scalding coffee). Instead, such models always predict the smallest possible trajectory deviation that does not contact the obstacle (with no ‘room for error’, so to speak). Moreover, the approach confounds the effect on trajectory of hitting an impenetrable obstacle and the cost to the subject. To return to the example we began with, it is easy to imagine circumstances where one would smash through the coffee cup to grasp something on the other side, such as a child in danger of falling. We see that obstacle avoidance, when viewed from the standpoint of Bayesian decision theory, can explain the amount of deviation around a virtual obstacle based on the cost of accidentally intersecting it, and the visuo-motor uncertainty in predicting the location of the fingertip when it passes the obstacle and when it reaches the target.

## Supporting Information

Figure S1 QQ-plots. Quantiles of horizontal fingertip position at the obstacle (a) and target (b) planes plotted against quantiles of a standard Gaussian distribution, for each of the 9 conditions. Data were normalized prior to plotting. Gaussian-distributed data would fall on a straight line.
(EPS)

Figure S2 Average excursions over the course of a block. Excursions are averaged over all blocks and subjects (the overall mean was set to zero). Excursion values remain approximately constant across a block; i.e., there does not appear to be any learning. In particular, subjects do not appear to adopt a strategy based on making initially large excursions, and subsequent ‘homing in’ on a final value.
(EPS)

Text S1 Model comparison. Basis for comparison of unity-line vs. non-unity-line models of the data.
(DOC)
