# Supplementary material for: Speeded Reaching Movements around Invisible Obstacles
Source: PLoS Comput Biol. 2012 Sep 20;8(9):e1002676. doi: 10.1371/journal.pcbi.1002676 (PMC3447970; doi:10.1371/journal.pcbi.1002676)
Supplement: Text S1 — Model comparison. Basis for comparison of unity-line vs. non-unity-line models of the data. (DOC) [file pcbi.1002676.s003.doc]

**Model Comparison**

Logically, a statistical comparison (i.e., a *t-test*) of the best-fitted line vs. a line of unity slope does not answer the question of whether results conform to the Bayesian hypothesis, because the parameter space of the Bayesian hypothesis (i.e., unity slope) is infinitesimal compared to the parameter space of the ‘alternative’ hypothesis (all non-unity slopes and nonlinear functional relationships). To correctly examine the Bayesian hypothesis, we must account for this discrepancy in the sizes of the two hypothesis spaces in comparing the two models.

As we have done previously[1,2,3,4], we perform the desired model comparison by computing a log-odds ratio based on the likelihood of each model:

and

.

Note that both model likelihoods are marginal probabilities, marginalizing over all model parameters (here , the possible slope values, and , an early-noise parameter defining the spread of observed average excursions from the fitted line – see below), where the probability of observing the *ij*^th^ datum (corresponding to the *i*^th^ obstacle and *j*^th^ value condition):

,

and the model variable ( = or ) simply restricts the possible values of , such that under , , and under , . Further, we assume Weber noise, such that the standard deviation describing expected deviations from the model line at the *ij*^th^ predicted shift, , is proportional to : . The leading term, , is a constant early-noise component.

Having no initial preference for either model, , the final log-odds ratio favoring the Bayesian model is obtained by combining (1) and (2):

,

which provides a *measure of evidence* [5] for the optimal model, expressed in dB.

References

1. Hudson TE, Landy MS (2012) Measuring adaptation with a sinusoidal perturbation function. J Neurosci Methods 208: 48-58.

2. Hudson TE, Maloney LT, Landy MS (2007) Movement planning with probabilistic target information. J Neurophysiol 98: 3034-3046.

3. Hudson TE, Maloney LT, Landy MS (2008) Optimal compensation for temporal uncertainty in movement planning. PLoS Comput Biol 4: e1000130.

4. Hudson TE, Tassinari H, Landy MS (2010) Compensation for changing motor uncertainty. PLoS Comput Biol 6: e1000982.

5. Jaynes ET, Bretthorst GL (2003) Probability theory: The logic of science. Cambridge, UK ; New York, NY: Cambridge University Press. xxix, 727 p.
